# Pixuna virus modifies host cell cytoskeleton to secure infection

**DOI:** 10.1038/s41598-017-05983-w

**Published:** 2017-07-18

**Authors:** Pedro Ignacio Gil, Guillermo Albrieu-Llinás, Estela Cecilia Mlewski, Marina Monetti, Laura Fozzatti, Cecilia Cuffini, José Fernández Romero, Patricia Kunda, María Gabriela Paglini

**Affiliations:** 1 Instituto de Virología “Dr. JM Vanella”, Facultad de Ciencias Médicas, Universidad Nacional de Córdoba, Córdoba, Argentina; 2 Center for Biomedical Research, Population Council, New York, USA; 3 Science Department, Borough of Manhattan Community College, New York, USA; 4 Centro de Investigaciones en Ciencias de la Tierra CICTERRA, CONICET, Universidad Nacional de Córdoba, Córdoba, Argentina; 5 Instituto de Investigaciones en Ciencias de la Salud (INICSA-CONICET), Facultad de Ciencias Médicas, Universidad Nacional de Córdoba, Córdoba, Argentina; 6 Instituto de Investigación Médica Mercedes y Martín Ferreyra, INIMEC-CONICET-Universidad Nacional de Córdoba, Córdoba, Argentina

## Abstract

Pixuna virus (PIXV) is an enzootic member of the Venezuelan Equine Encephalitis Virus complex and belongs to the New World cluster of alphaviruses. Herein we explore the role of the cellular cytoskeleton during PIXV replication. We first identified that PIXV undergoes an eclipse phase consisting of 4 h followed by 20 h of an exponential phase in Vero cells. The infected cells showed morphological changes due to structural modifications in actin microfilaments (MFs) and microtubules (MTs). Cytoskeleton-binding agents, that alter the architecture and dynamics of MFs and MTs, were used to study the role of cytoskeleton on PIXV replication. The virus production was significantly affected (p < 0.05) after treatment with paclitaxel or nocodazole due to changes in the MTs network. Interestingly, disassembly of MFs with cytochalasin D, at early stage of PIXV replication cycle, significantly increased the virus yields in the extracellular medium (p < 0.005). Furthermore, the stabilization of actin network with jasplakinolide had no effect on virus yields. Our results demonstrate that PIXV relies not only on intact MTs for the efficient production of virus, but also on a dynamic actin network during the early steps of viral replication.

## Introduction

Pixuna virus (PIXV) is a New World alphavirus from the family Togaviridae and belongs to the Venezuelan Equine Encephalitis (VEE) complex^1^. The VEE complex comprises 14 varieties of enzootic and epizootic viruses. The epizootic strains emerge periodically in regions of Central America and northern areas of South America (Venezuela and Colombia), causing outbreaks that involve humans and horses, with high morbidity and mortality rates^2, 3^. Mosquitoes with PIXV bite and infect birds, rodents, domestic animals and humans. Although PIXV was first isolated from *Anopheles* (*Stethomyia*) *nimbus* mosquitoes in Brazil, in 1961^4^ and has been detected later in Argentina^5^, little is known about the mechanisms concerning the cell biology of viral infection.

Viruses use the host cell machinery for entry, replication, transport, and release of viral progeny. After binding to receptors on the plasma membrane, alphaviruses enter the host cells by clathrin-mediated endocytosis and fusion with endosomal membranes^6^. Many studies have previously demonstrated that viruses can use actin microfilaments (MFs) and microtubules (MTs) transport systems to accomplish several steps during their life cycle^7–11^. These steps include: (i) the transport of newly synthesized viral proteins and genomes to specific sites within the infected cell for virus assembly and (ii) the membrane extrusion (viral budding) that ends with the exit of new viral progeny from the infected cell^7–11^. In the cell periphery and possibly in the nucleus, transport is mediated by the actin system, either by newly polymerized MFs that push the virus particles, or by myosins moving alongside actin filaments. The motor proteins dynein and kinesin catalyze transport along MTs, thus bridging the gap between the periphery and the cell center^12, 13^. In a later stage, the viral glycoproteins are transported in vesicles on MTs from the *trans* Golgi network to the site of budding beneath the plasma membrane prior to virus egress. Although some mechanism of trafficking, implicated in the modulation of alphavirus infection, have been elucidated^14^, little is known about the participation of cytoskeleton during the replication of New World alphaviruses.

In this study, we evaluated whether proper dynamics of MTs and MFs are necessary for a successful PIXV replication. To this end, we used chemical agents that interfere with the normal polymerization or depolymerization of MFs and MTs during different stages of PIXV replication cycle. Our results showed that infection with PIXV affected drastically the morphology of infected cells by altering the regular arrangement of cytoskeleton. Surprisingly, actin disruption during the early stage of viral replication increased significantly extracellular viral yields, while perturbations of MTs network strongly decreased virus production. Understanding the relationship of PIXV with the host cytoskeleton will provide not only a valuable insight into host cell-virus interactions, but may also explain the pathogenic properties of VEE viruses.

## Results

### The PIXV replication cycle

**Figure 1:**
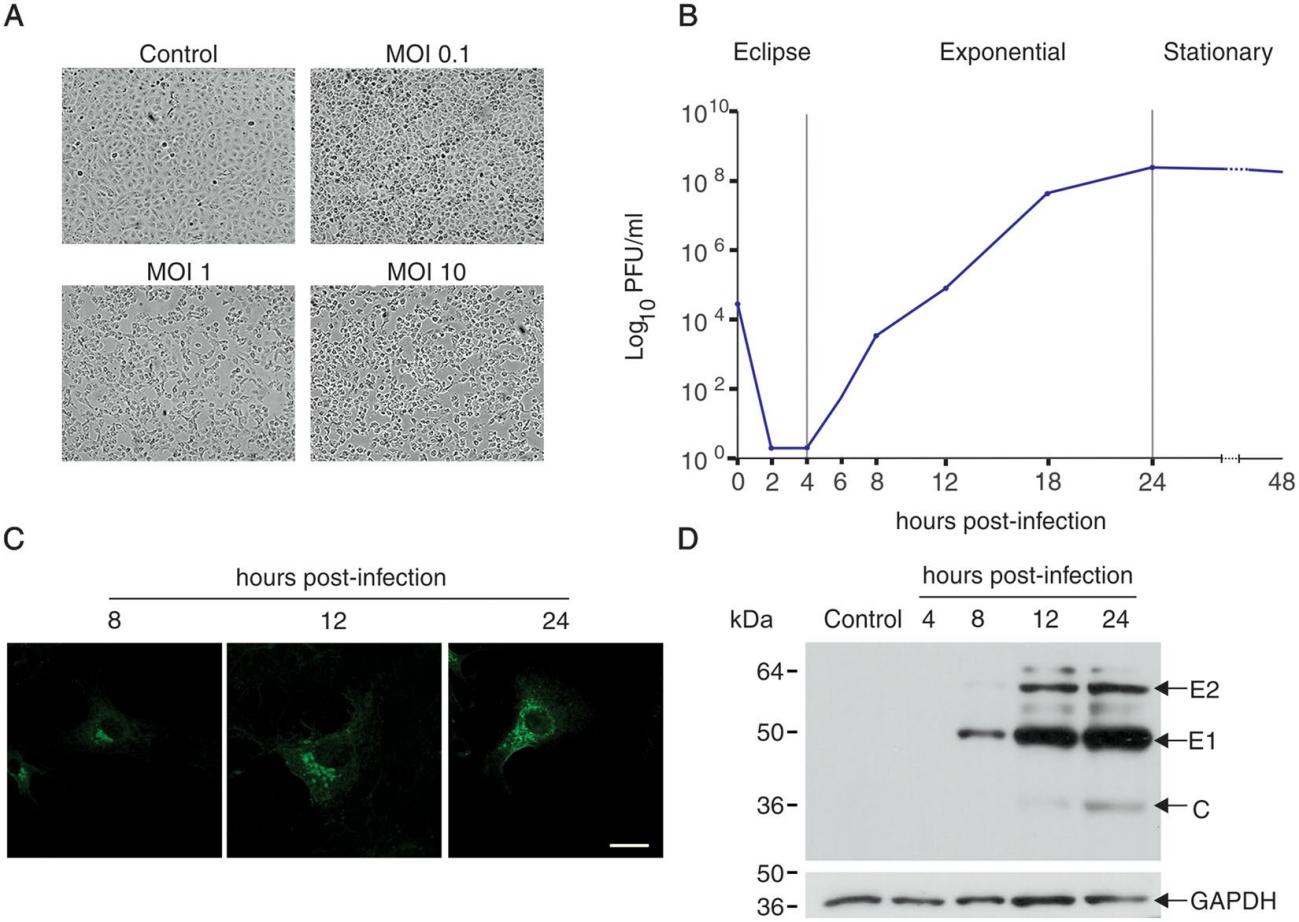
PIXV replication cycle in Vero cells. (**A**) PIXV induces modifications in the morphology of Vero cells at different MOI (0.1, 1 and 10) after 24 h of infection. Vero cells monolayer mock-infected or infected at MOI 0.1 (top) and MOI 1 or 10 (lower panel of the figure) were imaged with phase contrast microscopy at low magnification. The cytopathic effect (CPE) is observed from MOI 0.1, with the maximum disruption of the monolayer at MOI 10 (arrows). (**B**) PIXV growth curve in Vero cells. Monolayers were infected with MOI 0.1 and culture media were collected at different times (2, 4, 6, 8, 12, 18, 24 and 48 h.p.i.) and then assayed for infectious viral particles using a standard plaque assay. Note that infectious virus was detected after 12 h.p.i. and the maximum viral release was at 24 h.p.i. (**C**) Distribution of PIXV structural proteins during Vero cells infection. Vero cells were fixed at 8, 12 and 24 h.p.i. and processed for IF microscopy using an anti-PIXV mouse antibody and then Alexa Fluor 488-conjugated goat anti-mouse IgG (green). Scale bar = 20 µm. (**D**) Representative immunoblot showing the expression levels of PIXV structural proteins in infected Vero cells. Infected cells were harvested after 4, 8, 12 and 24 h p.i. Note that anti-PIXV polyclonal antibody recognizes E2, E1 and C viral proteins and that the levels of these proteins increase after 8 h p.i. E1: Envelope protein 1; E2: Envelope protein 2; C: Capsid protein. GAPDH was used as a loading control.

In order to characterize the different phases of the viral growth curve, the amount of infectious virus produced over time was evaluated by means of plaque assay. Vero cells were first infected with different multiplicity of infection (MOI = 0.1, 1, and 10) to select the proper MOI for subsequent experiments. After 24 h post infection (h.p.i.), monolayers infected with MOI = 0.1 showed a strong cytopathic effect, clearly identified under phase contrast microscope as cell rounding and cytoplasm enlargement. Moreover, monolayers infected with MOI = 1 and MOI = 10 showed an evident loss of confluence and severe cell damage (Fig. 1A). Based on these observations, the subsequent experiments were performed using MOI = 0.1. Figure 1B shows the duration of the different phases of PIXV growth curve. There was a lack of infectious virus in the extracellular medium during the first 4 h.p.i. (eclipse phase). The maximum viral yields were observed in the exponential or logarithmic phase that lasted 20 h. Finally, the infected cell monolayers reached their stationary phase, showing signs of lysis and cell death. The time course of PIXV infection was also studied using indirect immunofluorescence (IF) and Western blot. The presence of viral proteins was first visualized by IF at 8 h.p.i. in the perinuclear region, and after 12 h.p.i. in a large area of the cytoplasm (Fig. 1C). Western blot analyses identified PIXV viral proteins and showed increasing levels of viral protein expression during the course of infection. The viral protein E1 (49kD) was detected at 8 h.p.i., and subsequently E2 (58kD) and C (30kD) (Fig. 1D).

### The effects of PIXV infection on host cell cytoskeleton

**Figure 2:**
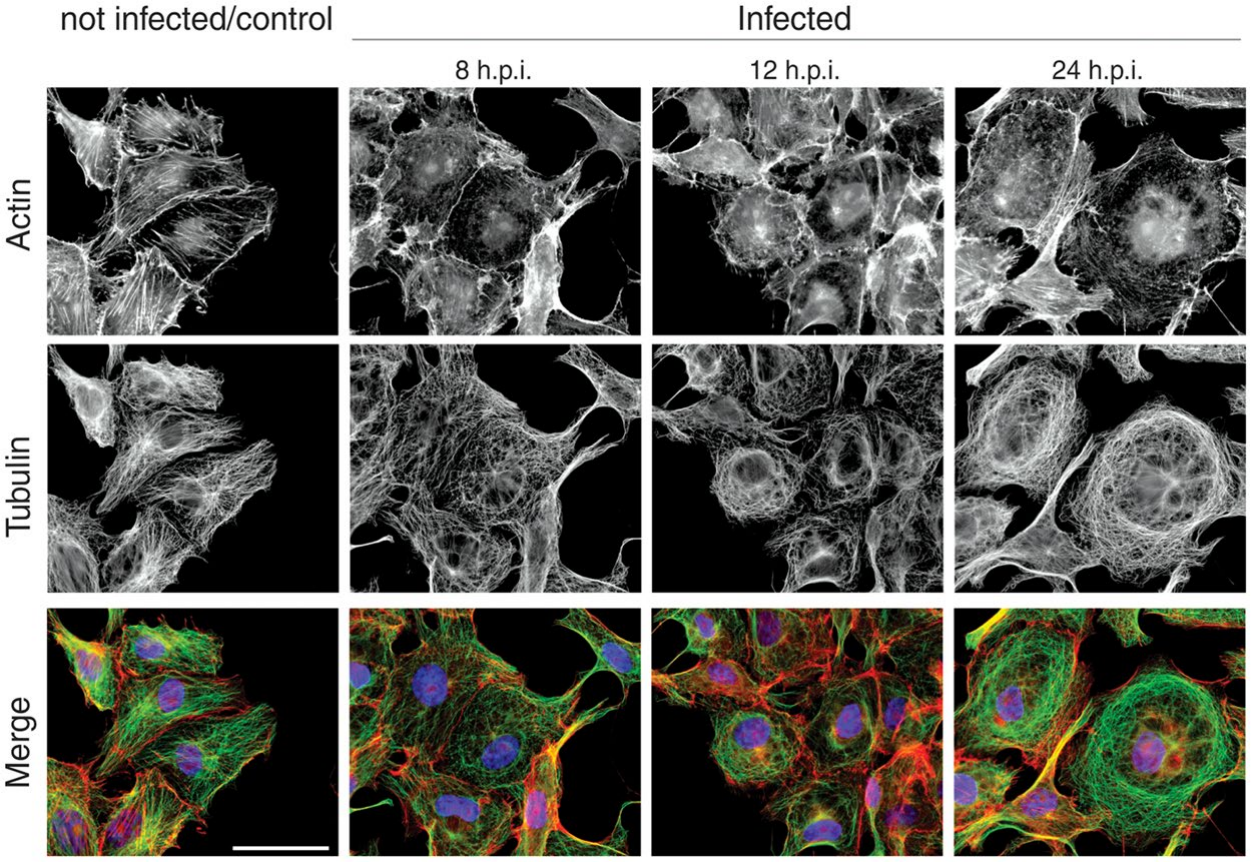
Time course changes in actin and tubulin cytoskeleton of PIXV-infected Vero cells. Mock-infected cells or cells infected with PIXV (MOI 0.1), were fixed at 8, 12 and 24 h p.i. and processed for IF. Actin and tubulin were visualized using phalloidin-rhodamine (red) and anti-α-tubulin antibody (green), respectively. Nuclei were stained with DAPI (blue). Note the progressive disorganization of cytoskeleton in infected cells after 8 h p.i. in comparison with control non infected cells. Scale bar: 20 µm.

We examined the impact of PIXV infection on the host cell cytoskeleton. For this purpose, Vero cell monolayers were fixed at 8, 12 and 24 h.p.i. and processed for IF. PIXV infection induced changes in MFs and MTs organization that affected the cell morphology (Fig. 2). At 8 h.p.i., MTs showed lost of the radial assembly from the microtubules organizing center, to distribute gradually in a concentric pattern towards the center of the cell. At 24 h.p.i. cells showed clear signs of enlargement and the MTs bundles were distributed in a concentric belt in the cell periphery (Fig. 2, middle panel). A similar feature was observed in the actin cytoskeleton, which showed an acute disruption after 8 h.p.i. MFs rearrangement was more evident after 12 h.p.i., and progressed over time until the actin fibers disappeared (Fig. 2, upper panel).

### The role of the host cell cytoskeleton during PIXV infection

**Figure 3:**
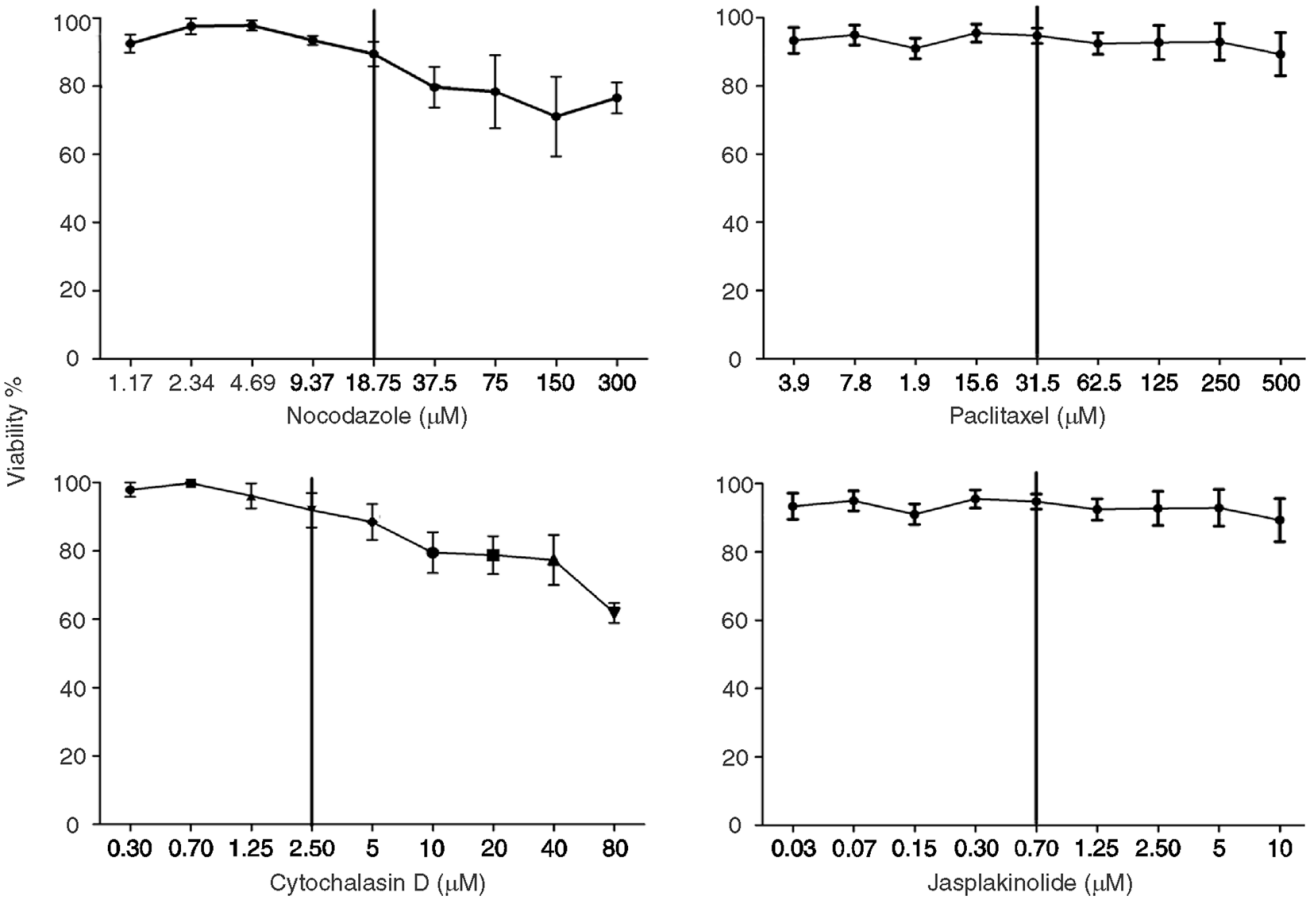
Dose-response curves to NZ, TX, Cyt D, and Jpk in Vero cells. The cellular metabolic activity was measured in Vero cells using the XTT assay. The graphs show % of cellular metabolic activity (mean ± SEM) relative to cell control (triplicates per condition, three independent experiments). The vertical lines represent non-toxic drug concentrations (90% of metabolic activity) chosen for subsequent assays.

The Vero cell monolayers were exposed to different concentrations of drugs that cause either stabilization or destabilization of MTs and MFs. The relative cellular metabolic activity after 24 h exposure was evaluated using the XTT assay. The concentration (for each drug) that caused only a 10% reduction in metabolic activity was used in subsequent experiments (Fig. 3). Table 1 summarizes the result of this experiment.

**Table 1:** Summary of drugs targeting the cytoskeleton and their mode of action.

Target	Drugs	Used Concentration (μM)	Mode of Action
**Microfilaments**	**Cytochalsin D**	**2.5**	Binds to filamentous actin and disrupts polymerization^32^
	**Jasplakinolide**	**0.7**	Binds to filamentous actin and stabilizes^33^
**Microtubules**	**Nocodazole**	**18.75**	Binds to β-tubulin and prevents interchain bonds^34^
	**Paclitaxel**	**31.5**	Binds to assembled microtubules and enhances the formation of highly stable microtubules^35^

**Figure 4:**
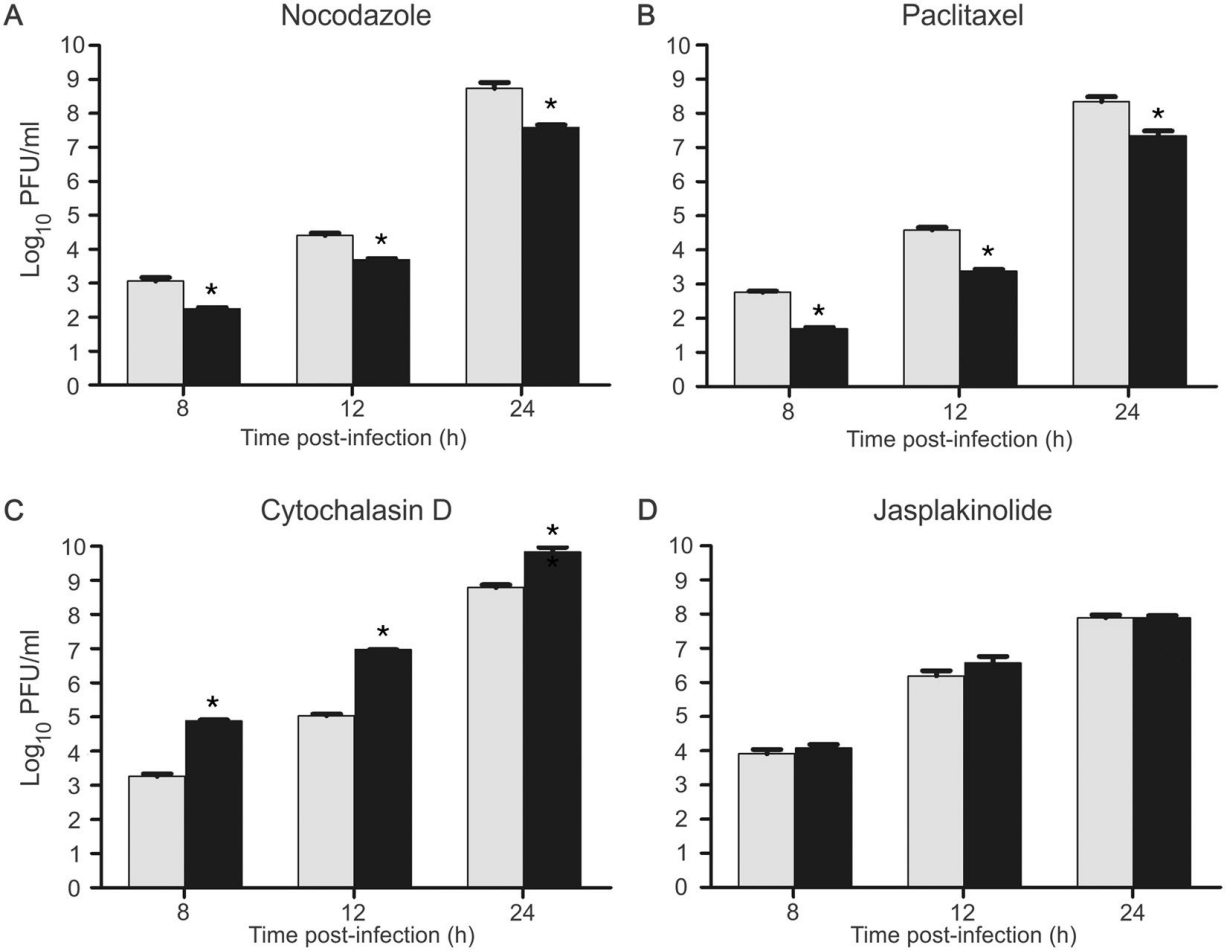
Effect of microtubule or microfilament disrupting agents on virus production during the entire viral cycle. Treated Vero cells (black bars) and control cells (grey bars) were infected with PIXV. Treated cells were exposed to NZ (**A**), TX (**B**), Cyt D (**C**) and Jpk (**D**) during the complete viral cycle. Infectious particles from the supernatants were quantified by plaque assay (pfu/ml) after 8 h, 12 h, and 24 h.p.i. The graphs show mean ± SEM (triplicates per condition, three independent experiments). Significant differences between treated and control cells are marked with * (p < 0.05).

To determine the role of MTs on the progression of viral cycle, Vero cells were infected with PIXV and continuously exposed to nocodazole (NZ; (18.75 μM) or paclitaxel (TX; (31.5 μM). The virus production was tested at 8, 12, and 24 h.p.i. by using the plaque assays. The treatment with NZ or TX produced a significant decrease in viral titers at every times point tested (Fig. 4A and B). NZ induced a 6-fold reduction in virus yields at 8 h.p.i. [F(1,10) = 9.145; p < 0.013], 5-fold at 12 h.p.i. [F(1,10) = 29.228; p < 0.001] and 14-fold at 24 h.p.i. [F(1,10) = 33.400; p < 0.001] when compared to their respective virus control (Fig. 4A). Treatment with TX showed a stronger effect than NZ, producing a 12-fold reduction in viral release at 8 h.p.i. [F(1,10) = 122.35; p < 0.001], 16-fold at 12 h.p.i. [F(1,10) = 8.026; p < 0.018] and 10-fold at 24 h.p.i. [F(1,10) = 4.965; p < 0.05] (Fig. 4B).

**Figure 5:**
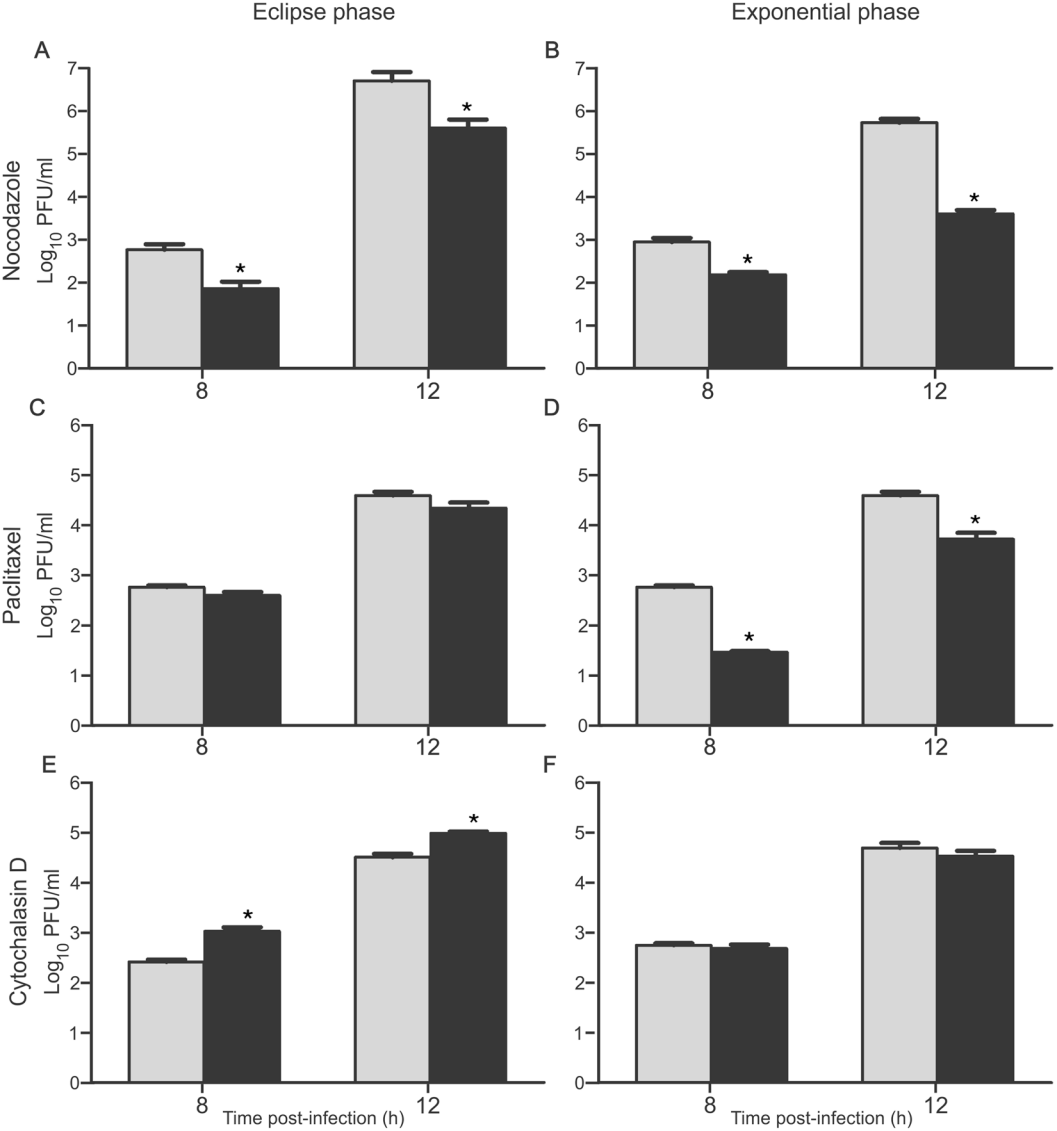
Effect of microtubule and microfilament disrupting agents on virus production at the different phases of the virus replication cycle. Cells were infected with PIXV and treated during the first 4 h.p.i. (eclipse phase **A**, **C** and **E**), or 4 to 12 h.p.i (exponential phase B, D and F) with NZ (**A,B**), TX (**C,D**) and Cyt D (**E,F**). Infectious particles in cell culture supernatants were quantified by plaque assay (pfu/ml) at 8 and 12 h.p.i. The graphs show mean ± SEM (triplicates per condition, three independent experiments). Significant differences between treated and control cells are marked with * (p < 0.05).

These results encouraged us to explore if MTs dynamic integrity has a functional impact during the early and/or late phases of PIXV replication. To this end, infected Vero monolayers were exposed to NZ or TX during the first 4 h.p.i (eclipse phase) or during the exponential growth phase, (4 to 12 h.p.i). The cell supernatants were harvested at 8 and 12 h.p.i. and the virus titers determined using the plaque assay. The treatment with NZ during the eclipse period induced a 8-fold reduction in virus yields at 8 h.p.i. [F(1,10) = 5.449; p < 0.042] and 9-fold at 12 h.p.i. [F(1,10) = 31.014; p < 0.001], when compared to the respective virus control (Fig. 5A). During the exponential phase, NZ caused a 6-fold reduction in the virus yields at 8 h.p.i. [F(1,10) = 11.667; p. < 0.001] and 129-fold at 12 h.p.i. [F(1,10) = 20.033; p < 0.001] (Fig. 5B). In contrast, no significant differences were observed in the viral titers after the treatment with TX during the eclipse period (Fig. 5C). Nonetheless, the treatment with TX during the exponential phase induced a fall of approximately 20-fold in the amount of extracellular infectious virus at 8 h.p.i. [F(1,10) = 133.44; p < 0.001] and 7-fold at 12 h.p.i. [F(1,10) = 21.599; p < 0.001] (Fig. 5D).

To further investigate the role of actin cytoskeleton on PIXV replication, monolayers of infected Vero cells were exposed to cytochalasin D (Cyt D; 2.5 μM) or jasplakinolide (Jpk; 0.7 μM). The treatment with Cyt D remarkably increased virus yields at every time point tested (Fig. 4C). By 8 h.p.i., cells treated with Cyt D produced a 40-fold increase in virus yields when compared to virus control [F(1,13) = 11.507; p < 0.005], 84-fold at 12 h.p.i. [F(1,12) = 36316; p < 0.001] and 11-fold at 24 h.p.i. [F(15,13) = 11.161; p < 0.005] (Fig. 4C). Otherwise, no significant changes were detected after exposure to Jpk at any time point (Fig. 4D).

To assess if the integrity of MFs dynamic has a functional impact during early and/or late phases, infected Vero monolayers were exposed to Cyt D during the first 4 h.p.i (eclipse phase) or during the exponential growth phase (4 to 12 h.p.i). The treatment with Cyt D during the eclipse period elicited a 4-fold increase in virus yields when compared to the untreated infected monolayers (virus control) at 8 h.p.i. [F(1,12) = 13.350; p < 0.003] and 3-fold at 12 h.p.i. [F(1,14) = 43.441; p < 0.001] (Fig. 5E). No significant changes in viral titers were detected after exposure to Cyt D during exponential phase of PIXV replication cycle (Fig. 5F).

## Discussion

The main goal of this study was to evaluate the interconnection between the host cell cytoskeleton and PIXV in the course of viral replication. To this end, we characterized the kinetics of PIXV growth, *in vitro*, through different methodologies, including plaque assay, IF microscopy and western blot. PIXV growth curve consisted in an eclipse phase that lasted 4 h, followed by 20 h of exponential phase during which the release of viral particles to the extracellular medium was accelerated. The growth curve we obtained in Vero cells is consistent with the one we previously described in mouse embryonic fibroblasts^15^. The kinetic of PIXV proteins, detected by IF and western blot, are also in agreement with the viral growth curve (Fig. 1).

The strong cytopathic effect, identified under phase contrast microscope as cell rounding and cytoplasm enlargement, was most likely due to the noticeable reorganization of the cytoskeleton. In fact, MFs were disrupted in the subcortical region of infected cells, and they became more disorganized towards the end of PIXV replication cycle (Fig. 2). Additionally, MTs also lost their typical arrangement and, at 24 h.p.i., they were clearly distributed concentrically around the nucleus (Fig. 2). These observations are in agreement with previous reports that demonstrated that other alphavirus proteins dismantle or reorganize the MTs and MFs. In this sense, a proteomic analysis and functional studies have revealed that cytoskeleton proteins, including actin and tubulin, are regulated differentially upon Chikungunya (CHIKV) infection. Interestingly, actin was found to be down-regulated after infection with CHIKV in SJCRH30 rhabdomyosarcoma cells^16^. Moreover, an intact actin network is essential at the early stages of Semliki Forest virus (SFV, alphavirus) trafficking and its disruption has been observed at the late time-points of infection^17^. This indicates that MFs may play an important role mediating viral trafficking in the alphavirus family.

Subsequently, the possible role of the cytoskeleton during different stages of PIXV replication cycle was analyzed using drugs that alter the normal architecture and dynamics of MTs and MFs. Treatment with TX or NZ during the entire cycle caused a significant decrease in virus yields. This suggests that the stabilization of MTs and/or the inhibition of its polymerization affect the intracellular viral kinetics. The destabilization of MTs with NZ during the eclipse or during the exponential phase had an inhibitory effect on the release of viral particles. We have to consider that NZ has a reversible destabilizing effect on MTs, meaning that these structures recover their normal dynamics once the drug is removed. In this context, it is remarkable that an early destabilization of MTs, during only the first 4 hours, is sufficient to reduce the virus release at different time points after infection. Contrarily, the stabilization of MTs with TX must be sustained throughout the replication cycle to obtain a similar result; besides, the treatment with TX seems to have a milder effect on the release of infective particles. Taken together, these results suggest that intact MTs are essential for the maturation and/or trafficking processes of PIXV proteins to specific sites. This process is performed to achieve an effective viral assembly and budding of viral progeny, as supported by previous studies with other viruses^8, 13, 18, 19^. Frolova *et al*.^20^ observed that NZ significantly reduces the budding of another alphavirus, Sindbis virus (SINV). This study suggests that NZ blocks the release of virus particles by inhibiting cytopathic vacuoles and/or glycoprotein transport instead of interfering with viral RNA replication. Additionally, the movement of SFV replication complexes is not required for RNA replication and virus production, but MTs disruption by NZ has a negative effect on virus release^17^.

We then studied the role of actin on PIXV infection, using drugs that alter the structural integrity and dynamics of MFs. The treatment with Cyt D increased the virus yields in the extracellular medium. Furthermore, the actin-stabilizing agent, Jpk, had no effect over virus yield. These results indicate that the disruption of the actin cytoskeleton could facilitate the entry of viral particles into the host cell, probably promoting endocytosis and thus increasing the entry of viral particles into the endosomal system. It is well known that actin assembly plays an essential role in several processes leading to endocytosis, including the internalization of clathrin-coated vesicles^21^. Since alphaviruses enter the target cells by clathrin-mediated endocytosis^22, 23^, it is reasonable to think that the cortical actin barrier underlying the plasma membrane might need to be dissolved or removed, as proposed by Qualmann *et al*.^24^. Regarding the effect of MFs-binding agents on virus yields, it has been reported that Cyt D does not reduce SINV yields in infected cells^25^. However, the exposure to the same drug significantly reduces the percentage of cells infected with CHIKV, suggesting that the integrity of actin fibers is required at least for the internalization of this virus^26^.

In summary, our findings demonstrate an increase in extracellular viral titters after the disorganization of actin MFs with Cyt D. This particular observation, together with the remarkable reorganization of subcortical actin, raises new questions about possible interactions between PIXV and MFs, as well as particular requirements of cytoskeleton dynamics during viral entry. More importantly, we observed that either the inhibition of polymerization of MTs with NZ or their stabilization with TX interfere with PIXV infection cycle in host cells. This indicates that specific interactions with these elements are necessary for the normal development of viral replication. Considering that these drugs are already used in human chemotherapy^27^, it would be interesting to study their potential antiviral activity.

Although the precise mechanisms of interaction between PIXV and the host cell cytoskeletal elements remain to be defined, knowing the different interactions between this alphavirus and cytoskeletal components is crucial, not only for understanding the basis of early pathogenesis, but also for shedding light on possible new antiviral treatments.

## Experimental Procedures

### Cells and virus

Vero cells (Vero 76, ATCC CRL 1587) were grown in Dulbecco’s modified Eagle’s medium (DMEM) (GIBCO, USA) with 10% fetal calf serum (FCS), Gentamicin (50 µg/ml), at 37 °C, 5% CO_2_ and 98% humidity. The PIXV strain BeAr 35645, isolated from *Anopheles nimbuspor* in Brazil^4^, was propagated in Vero cells and stocks were stored at 7 × 10^6^ plaque-forming units (PFU/ml). Virus yields (PFU/ml) were determined by a standard plaque assay^28, 29^. Briefly, infected Vero cell monolayers were overlaid with 1.5% ultra pure agarose (Gibco BRL) in 4% DMEM 2x. Two days later, the cells were fixed with 10% formaldehyde in water and the overlaid medium was removed. Then, the monolayers were stained with 1% crystal violet in water and the plaques were counted. Infectivity titers were expressed as PFU/ml. All the experiments with PIXV were performed in a laminar flow hood under biosafety level 2 conditions.

### Synchronized infection

Vero cells were plated at 1 × 10^5^ cells/well in 100 μl of complete medium and incubated at 37 °C, 5% CO_2_ and 98% humidity. Confluent monolayers of Vero cells were infected at different multiplicity of infection (MOI) 0.1, 1 or 10. Virus adsorption was performed at 4 °C per 1 h. After this first incubation, the inoculum was removed and monolayers were washed with phosphate-buffered saline (PBS) at 4 °C to eliminate any free virus particle and DMEM with 1% FCS was added before incubating at 37 °C, 5% CO_2_ and 98% humidity for 8, 12 or 24 h, depending on the experiment. Cytopathic effect was evaluated by direct visualization by phase contrast microscopy at low magnification, using an Olympus IX81 microscope.

### Time course of infection

After identifying a MOI of 0.1 as the optimal MOI to obtain cytopathic effects without compromising the integrity of the cell monolayer, Vero cells were infected at this MOI as described in the above section. Supernatants were harvested at 2, 4, 6, 8, 12, 18, 24 and 48 h post infection (h.p.i.) and tittered by plaque assay.

### IF staining

To visualize the actin and microtubule network, cell monolayers were grown at 60% confluence on glass coverslips, washed three times with PBS, fixed with 4% paraformaldehyde/4,1% sucrose (Riedel-de Haën, Sigma-Aldrich Laborchemikalien GmbH, Seelze, Germany) for 20 min at room temperature (RT) and then washed with PBS. The cells were then permeabilized with 0.2% Triton^TM^ X-100 (Sigma-Aldrich, St. Louis, MO) in PBS for 5 min at RT and incubated with 5% bovine serum albumin (BSA, Sigma-Aldrich, St. Louis, MO) for 1 h at RT. The cells were incubated with the primary antibodies (mouse anti-α-tubulin – DM1A, Sigma-Aldrich, St. Louis, MO) diluted 1/1000 in 1% BSA/PBS solution over night at 4 °C. Then, monolayers were washed three times with PBS, and incubated with the secondary antibodies (goat anti-mouse, Alexa Fluor 488 (Thermo Fisher Scientific Inc., Waltham, Massachusetts, USA) for 2 h at RT. To visualize the actin filaments, the fixed and permeabilized cell monolayers were labeled with Phalloidin-Tetramethyl rhodamine B (Sigma-Aldrich, St. Louis, MO) for 1 h at RT. In order to evidence viral structural proteins, the same procedure was performed using an anti-PIXV primary polyclonal IgG antibody generated in mice, in our laboratory. Cells were visualized using a conventional inverted epi-fluorescence microscope (Olympus IX81, Olympus Corporation, Shinjuku, Tokyo). Images were processed using Adobe Photoshop CS6 (Version: 13.0).

### Western blot

Changes in the levels of intracellular viral proteins sequential expression were analyzed by western blot as previously described^30^. Briefly, infected Vero cells were lysed in RIPA buffer (1% Triton X-100, 0.5% deoxycholate, 0.1% SDS, 1 mM CaCl_2_, 0.5 mM MgCl_2_, and protease inhibitor cocktail) after 4, 8, 12, and 24 h.p.i., the insoluble debris was removed by centrifugation at 16,000 g. Forty μg of whole-cell extracts were resolved on 12% SDS-PAGE and transferred to polyvinylidene difluoride membranes (PVDF, Thermo Scientific, IL, USA). The membranes were incubated with anti-PIXV mouse primary polyclonal antibody (1/1000) overnight at 4 °C and then with a secondary horseradish peroxidase-conjugated anti-mouse antibody (1/800) (Promega Corporation, Madison, WI) for 1 h at RT. Anti-GAPDH antibody was used as a loading control (Sigma-Aldrich, St. Louis, MO, 1:5000). Blots were developed using a chemiluminiscence detection kit (ECL; GE Amersham) for the visualization of reactive bands.

### Pharmacological treatment

Cellular metabolic activity was evaluated after treatment with increasing concentrations of NZ (1–300 μM), TX (2–500 μM), Cyt D (0.30–80 μM) (Sigma-Aldrich, St. Louis, MO), and Jpk (0.03–10 μM) (Calbiochem, St. Diego, CA). Cellular metabolic activity was determined after 24 h of treatment using a colorimetric assay based on the reduction of tetrazolium salt (2,3-bis[2-methyloxy-4-nitro-5-sulfophenyl]-2H-tetrazolium-5-carboxanilide [XTT])^31^. A concentration of each drug that results in cellular metabolic activity higher than 90% was chosen for subsequent experiments (Table 1). Synchronized virus adsorption was performed at 4 °C as described above and monolayers were exposed to the different drugs during 24 h (complete cycle; NZ, TX, Cyt D, and Jpk) or during each viral cycle phase (eclipse and exponential growth phase up to 12 h; for NZ, TX and Cyt D). After the treatment period, the culture supernatants were harvested at 8, 12, and 24 h.p.i., and extracellular infective PIXV titers were determined by standard Vero cell plaque assay and expressed as plaque forming units per milliliter (PFU/mL).

### Statistical analysis

All data sets passed the normality test (D´Agostino –Pearson normality test). Data are representative of at least three independent experiments and values are given as mean ± standard deviation (SD). Statistical analysis was carried out using one-way ANOVA test, followed by post-hoc Fisher´s analysis to enable specific group comparison. A p < 0.05 was considered statistically significant. All tests were performed using Statistic 7 (Statsoft, Inc.; OK, USA).

### Data Availability

The datasets generated during and/or analysed during the current study are available from the corresponding author on reasonable request.
